# Peak appearance time in pulse waveforms of intracranial pressure and cerebral blood flow velocity

**DOI:** 10.3389/fphys.2022.1077966

**Published:** 2023-01-04

**Authors:** Arkadiusz Ziółkowski, Agata Pudełko, Agnieszka Kazimierska, Agnieszka Uryga, Zofia Czosnyka, Magdalena Kasprowicz, Marek Czosnyka

**Affiliations:** ^1^ Department of Biomedical Engineering, Faculty of Fundamental Problems of Technology, Wroclaw University of Science and Technology, Wroclaw, Poland; ^2^ Brain Physics Laboratory, Division of Neurosurgery, Department of Clinical Neurosciences, Addenbrooke’s Hospital, University of Cambridge, Cambridge, United Kingdom; ^3^ Institute of Electronic Systems, Faculty of Electronics and Information Technology, Warsaw University of Technology, Warsaw, Poland

**Keywords:** transcranial Doppler, cerebral blood flow velocity, morphological analysis, pulse shape analysis, traumatic brain injury, intracranial pressure, plateau waves, hypocapnia

## Abstract

The shape of the pulse waveforms of intracranial pressure (ICP) and cerebral blood flow velocity (CBFV) typically contains three characteristic peaks. It was reported that alterations in cerebral hemodynamics may influence the shape of the pulse waveforms by changing peaks’ configuration. However, the changes in peak appearance time (PAT) in ICP and CBFV pulses are only described superficially. We analyzed retrospectively ICP and CBFV signals recorded in traumatic brain injury patients during decrease in ICP induced by hypocapnia (*n* = 11) and rise in ICP during episodes of ICP plateau waves (*n* = 8). All three peaks were manually annotated in over 48 thousand individual pulses. The changes in PAT were compared between periods of vasoconstriction (expected during hypocapnia) and vasodilation (expected during ICP plateau waves) and their corresponding baselines. Correlation coefficient (r_S_) analysis between mean ICP and mean PATs was performed in each individual recording. Vasodilation prolonged PAT of the first peaks of ICP and CBFV pulses and the third peak of CBFV pulse. It also accelerated PAT of the third peak of ICP pulse. In contrast, vasoconstriction shortened appearance time of the first peaks of ICP and CBFV pulses and the second peak of ICP pulses. Analysis of individual recordings demonstrated positive association between changes in PAT of all three peaks in the CBFV pulse and mean ICP (r_S_ range: 0.32–0.79 for significant correlations). Further study is needed to test whether PAT of the CBFV pulse may serve as an indicator of changes in ICP–this may open a perspective for non-invasive monitoring of alterations in mean ICP.

## Introduction

**FIGURE 1:**
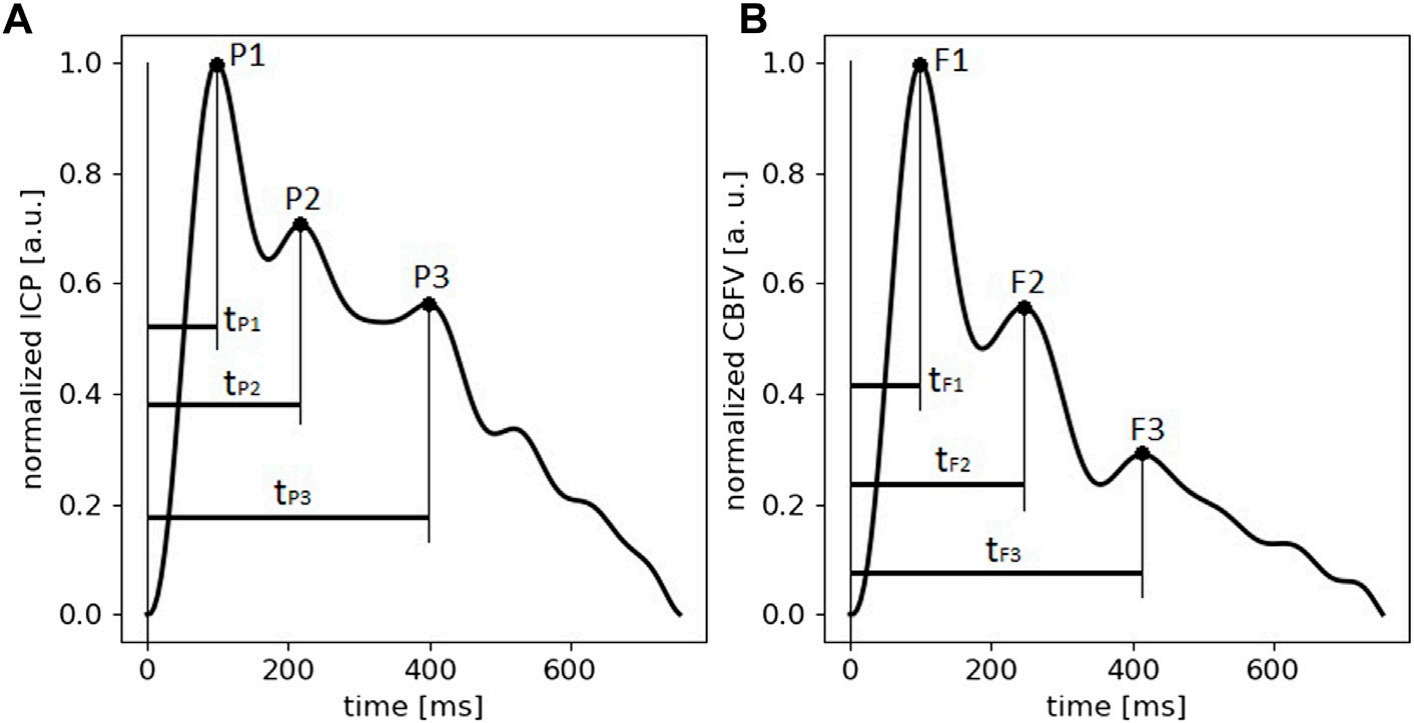
An example of normalized pulses of intracranial pressure (ICP) and cerebral blood flow velocity (CBFV) with annotated peaks (P1, P2, P3 for ICP pulse and F1, F2, F3 for CBFV pulse) and peak appearance times (t_Pn_–peak appearance time of the *n*th peak of ICP pulse, t_Fn_–peak appearance time of the *n*th peak of CBFV pulse).

Both intracranial pressure (ICP) and cerebral blood flow velocity (CBFV) signals have a pulsatile character due to pulsations of arterial blood pressure (ABP) and cerebral arterial blood volume (C_a_BV) during the cardiac cycle. In normal conditions, the ICP pulse contains three local maxima (called peaks, see Figure 1A). The first peak (P1) is associated with the systolic peak of ABP and CBFV pulses and the second (P2) with the maximum of C_a_BV pulse; the origin of the third peak (P3) is not well described (Carrera et al., 2010). However, it is hypothesized that P3 may be related to venous blood outflow (Czosnyka and Czosnyka, 2020). Changes in the shape of the ICP pulse waveform have been linked to rises in mean ICP (Chopp and Portnoy, 1980; Contant et al., 1995) or to reduced ability of the cerebrospinal system to compensate for volume increases (i.e., diminished cerebrospinal compensatory reserve) (Heldt et al., 2019). It is assumed that with higher ICP the buffering capacity of the system is compromised. However, at normal ICP, the compensatory reserve can also be affected due to the cerebrospinal fluid and cerebral blood volume already displaced from the intracranial space or brain lesions secondary to injury. At good compensatory reserve, P1 is usually the highest in comparison to the other two (P2 and P3) (Cardoso et al., 1983). Under pathophysiological conditions, with rise in mean ICP and/or reduction of intracranial compliance, initially the amplitudes of the first two peaks (P1 and P2) increase, then P2 becomes dominant (Germon, 1988). Based on this observation, the ratio of amplitudes of peaks P1 and P2 (P1/P2) was proposed to reflect intracranial compliance (Cardoso et al., 1988) and its usefulness to assess changes in status of compensatory reserve has been demonstrated (Kazimierska et al., 2021). Moreover, other studies showed that changes in ICP pulse peak configuration may reflect intracranial hypertension (Hamilton et al., 2009; Hu et al., 2010b) and may be associated with impaired cerebral autoregulation (Hu et al., 2010a) and cerebrovascular modulation (Asgari et al., 2011).

A typical CBFV pulse also exhibits three peaks (Kim et al., 2011) (see Figure 1B). The first peak (F1), linked with the ejection of blood from the left ventricle of the heart, is usually dominant. The second (F2) is related to blood pulse wave reflection from closing heart valves, and partially the Windkessel effect (releasing of the energy stored in elastic walls of vessels to the blood flow). The third peak (F3) is associated with diastolic blood flow (Aggarwal et al., 2008; Khan and Wiersema, 2020). The height of peaks F1 and F2 decreases with age and this decline is usually more pronounced for F1, meaning that the second peak may become dominant (Flück et al., 2014). The ratio of the first two peaks (F2/F1) is called the augmentation index (AI) (Wilkinson et al., 1998; Kohara et al., 2005). It was adopted from studies on the radial pressure pulse waveform and is considered an estimator of cerebrovascular stiffness (Kurji et al., 2006; Flück et al., 2014). The shape of the CBFV pulse also changes with alterations in mean ICP. It has been described in patients with acute liver failure that with ICP rise, the amplitude of F1 increases and the peak becomes more spiky, the diastolic flow phase becomes longer, and end diastolic velocity decreases (Aggarwal et al., 2008). As mean ICP continues to increase, the F2 peak disappears from the CBFV waveform, mainly due to external compression of the vessel wall leading to reduced vascular compliance and resulting in damping of the Windkessel effect (Aggarwal et al., 2008; Khan and Wiersema, 2020). Similar changes in the CBFV waveform were noticed in patients after traumatic brain injury (TBI) during moderate rises in ICP. When mean ICP increases, the amplitude of F3 decreases and the systolic part of the pulse (the F1 region) becomes more dominant (Kim et al., 2013), increasing TCD pulsatility index (De Riva et al., 2012).

While the parameters of ICP and CBFV waveforms related to peak height were investigated in a broad context (Aggarwal et al., 2008; Kim et al., 2013, 2011; Anile et al., 2014; Dias et al., 2014; Cardim et al., 2017; Kazimierska et al., 2021), peak appearance times (PATs; also called peak latency) were seldom studied and always as secondary indices complementing the analysis of peak heights. Nonetheless, in several studies using the Morphological Clustering and Analysis of Continuous Intracranial Pressure (MOCAIP) algorithm (Hu et al., 2009) PATs were suggested to be influenced by changes in cerebral hemodynamics (Hu et al., 2010a; Asgari et al., 2011; Kim et al., 2011). Therefore, in this work, we aimed to extensively analyze how the PATs of the ICP and CBFV pulses change during vasoconstriction (induced by hypocapnia) and vasodilation (accompanying ICP plateau waves) in TBI patients. Additionally, we aimed to investigate the relationship between PAT and changes in ICP during hypocapnia and ICP plateau waves.

## Methods

### Patient cohort

We analyzed retrospectively high frequency recordings of ICP, CBFV, and ABP signal from a database of 345 adult TBI patients hospitalized at Addenbrooke’s Hospital (Cambridge, United Kingdom) between 1992 and 2009. The patients were sedated and mechanically ventilated. Decreases in ABP resulting in reductions of cerebral perfusion pressure (CPP) below 60 mm Hg were managed with fluid loading and dopamine injections (2–15 μg kg^−1^ min^−1^), and if required, norepinephrine injections at a rate of 0.5 μg kg^−1^ min^−1^. If ICP rose above 20 mm Hg, boluses of mannitol were administered (200 ml of 20% solution over a period of 20 min or longer) and the ventilation volume was increased in order to achieve mild hypocapnia (partial pressure of arterial CO_2_ range: 28–35 mm Hg).

All signals were recorded as part of standard clinical procedures aimed at daily monitoring of cerebral autoregulation (Czosnyka et al., 2001). This form of multimodal brain monitoring was accepted by the multidisciplinary Neurosciences Critical Care Unit (NCCU) user committee before 1997. After 1997, regional ethical committee approval was obtained (30 REC 97/291) for anonymized data recording. The data was fully anonymized with no risk of any data protection issues and the study was conducted in accordance with the principles embodied in the Declaration of Helsinki.

### Data acquisition and monitoring

CBFV was monitored in the M1 segment of middle cerebral artery using transcranial Doppler ultrasonography systems Multi Dop X4 (DWL Elektronische Systeme, Sipplingen, Germany) or Neuroguard (Medasonic, CA, United States) with a 2-MHz probe. The side of TCD monitoring was the same as the side of ICP probe insertion, which was chosen individually for every patient. ICP was monitored using intraparenchymal probes (Codman & Shurtleff, MA, United States or Camino Laboratories, CA, United States). ABP was monitored invasively from the radial artery using pressure monitoring kits (Baxter Healthcare CA, United States; Sidcup, United Kingdom). All signals were recorded simultaneously with the sampling rate of 50 Hz using an analog-to-digital converter (DT9801, Data Translation, Marlboro, Mass, United States) and software for waveform recording (WREC, W. Zabolotny, Warsaw University of Technology, Poland) or BioSAn (University of Cambridge, United Kingdom) software. The end tidal CO_2_ level was controlled but was not monitored continuously.

#### Plateau waves

A total of 20 TBI patients with recorded ICP plateau waves were selected for the plateau wave group. Initial inclusion criterion was simultaneous recordings of ICP and CBFV signals during an episode of plateau wave. This criterion was the primary reason for the low number of identified patients as CBFV was monitored daily for periods of 10 min to 1 h and therefore, the probability of capturing a plateau wave within the limited time of CBFV monitoring was low. Median age of patients was 29 years (interquartile range (IQR): 20–44 years) and 25% of the group were female. Median Glasgow Coma Scale score at admission was 5 (range: 3–12). Monitoring of the CBFV signal was associated with a daily investigation of cerebral autoregulation (Czosnyka et al., 1999).

Plateau waves were identified post-recording during offline analysis with the following criteria: mean ICP elevation above 40 mm Hg, relative mean ICP increase greater than or equal to 15 mm Hg, and CPP decrease greater than or equal to 10 mm Hg for over 3 min. The definition of plateau wave followed the criteria from experimental work (Rosner and Becker, 1984) as closely as possible but taking into account the clinical management of prolonged ICP increases.

This material was analyzed in (Kim et al., 2009; De Riva et al., 2012; Varsos et al., 2013), however not in the context of peak appearance times.

#### Hypocapnia

29 TBI patients who underwent (based on clinical indications) a controlled mild decrease in PaCO_2_ with a simultaneous recording of ICP and CBFV signals were selected for the hypocapnia group (Puppo et al., 2020). Median age of the patients was 39 years (range: 17–70 years) and 21% were female. Median Glasgow Coma Scale at admission was 6 (range: 3–12). To perform CO_2_ change, the ventilator minute volume was increased by 15–20% after acquiring baseline data for 20 min. The lowest allowed PaCO_2_ was 26 mm Hg and the lowest allowed jugular bulb saturation was 55%. If these values were reached, hyperventilation was immediately stopped. A 10-min stabilization period was followed by a 50-min stable hyperventilation period during which the ventilator settings remained unchanged. PaCO_2_ values were determined by arterial blood gas analysis at the beginning and at the end of the stable hyperventilation period to validate end-tidal carbon dioxide values. The study protocol was presented prospectively to the multidisciplinary NCCU user committee to address ethical issues concerning the publication of these data which were routinely collected as a part of standard clinical monitoring. The same material was previously analyzed in (Steiner et al., 2004; Carrera et al., 2011; Puppo et al., 2020; Ziółkowski et al., 2021a), however not with reference to peak appearance times. We wish to thank Dr L. Steiner for sharing the material recorded as a part of his PhD project in Cambridge.

### Data analysis

#### Pre-processing

**FIGURE 2:**
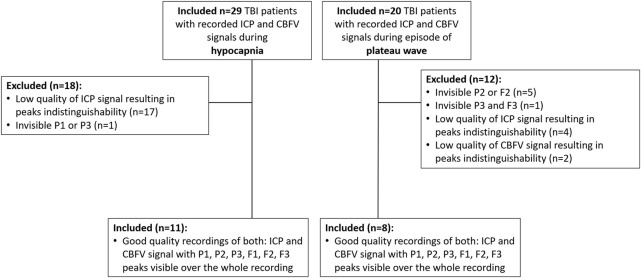
Flow diagram of the exclusion process performed to select viable signals with a recorded period of hypocapnia or ICP plateau wave.

Recordings from the preliminarily selected patient groups were visually inspected in ICM + software (Cambridge Enterprise Ltd., Cambridge, United Kingdom) to eliminate signals with insufficient quality or indistinguishable peaks in the ICP or CBFV pulses over the majority of the recording time. The exclusion process is presented in Figure 2.

Eight recordings with ICP plateau waves and 11 recordings during hypocapnia (each recording was taken from a different patient, with no overlapped data) were included in the final group. Two periods were manually selected from each recording for further analysis: approximately 15 min (or less if any gap in the signals occurred) of good quality signals preceding hypocapnia or ICP plateau wave (baseline period) and a minimum of 10 min of good quality signals during hypocapnia phase or the entire plateau wave (stable period). The baseline period was defined as the part of the recording before the change in ICP associated with hypocapnia or plateau wave occurred. The plateau phase was designated as the period in which mean ICP was greater than or equal to 90% of the maximal ICP value observed during the plateau wave. The hypocapnia period was selected from the recording when mean ICP decreased below 110% of minimal observed ICP value and ended either when mean ICP passed the 110% threshold again or the recording ended or the duration of the selected interval reached 800 s. Duration of selected analysis periods was affected mainly by the availability of non-disrupted recordings and the duration of each plateau waves. The distribution of these times is presented in the Results section.

All signals were up-sampled to the frequency of 200 Hz with simple linear interpolation to increase their temporal resolution and enable precise manual annotation of the peaks. In order to remove high-frequency noise from both ICP and CBFV signals, a low-pass filter with cut-off frequency of 12 Hz was applied. The cut-off frequency was selected based on previous analyses showing that the power of the ICP signal is mostly contained in the range below 8 Hz (Calisto et al., 2010). Individual pulse onset points were detected with modified Scholkmann algorithm (Bishop and Ercole, 2018) and the pulses were normalized to 0–1 range. Heart rate was calculated based on the duration of individual CBFV pulses: 1/duration. Pulse-by-pulse analysis of the ABP signal was not performed as it could be disturbed by the distance between measurement sites (ABP: peripheral measurement; CBFV and ICP: intracranial measurement).

#### Peak annotation

A single researcher (AP) annotated all three peaks in every reliable pulse of ICP and CBFV (P1, P2, P3 and F1, F2, F3, respectively) or marked the pulse as invalid if any of the three peaks was indistinguishable or the shape of the pulse was distorted. Distorted pulses were identified based on examples shown in previous studies on ICP and CBFV pulse waveforms (Lee et al., 2019; Megjhani et al., 2019; Ziółkowski et al., 2021b; Mataczynski et al., 2021) to exclude, among others, non-physiological signal values resulting from artifacts at the signal collection stage. The peak appearance times were then calculated for every reliable pulse as the time interval between the local minimum preceding the ascending slope of the pulse and the annotated position of a peak. An example of ICP and CBFV pulses with peak appearance times is visualized in Figure 1. The appearance times of the first, second, and third peak were named t_P1_, t_P2_ and t_P3_ for ICP pulses (Figure 1A) and t_F1_, t_F2_, t_F3_ for CBFV pulses (Figure 1B). The annotation process and calculation of all parameters were performed using programs custom-written in Python 3.7.

#### Analysis of changes in peak appearance times

**FIGURE 3:**
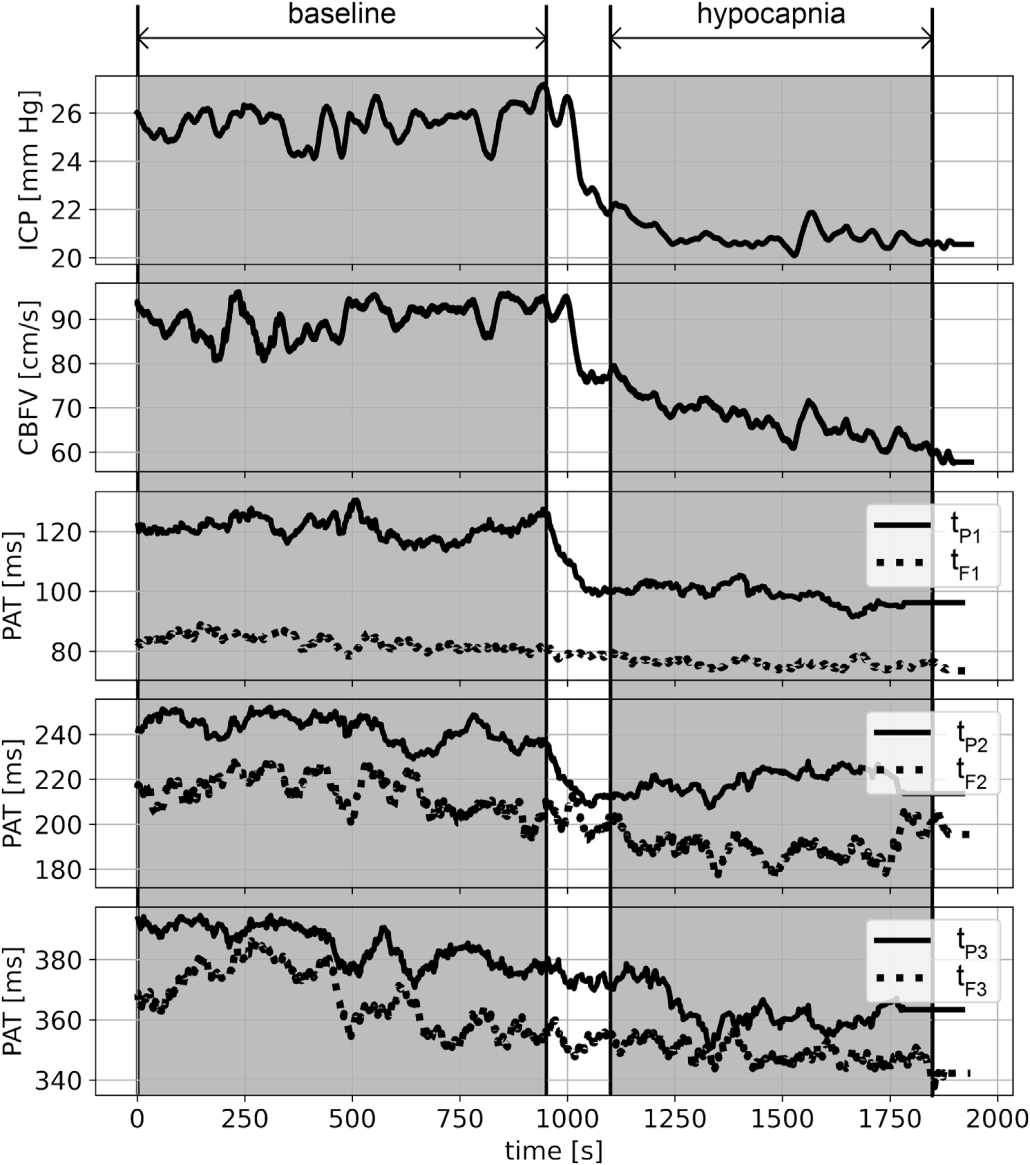
An example of smoothed (within the window of 40 pulses) recordings from a single patient during hypocapnia. From top to bottom: intracranial pressure (ICP), cerebral blood flow velocity (CBFV), peak appearance times (PATs) of the first, second, and third ICP peaks (t_P1_, t_P2_, t_P3_) and the first, second and third CBFV peaks (t_F1_, t_F2_, t_F3_). Shaded areas indicate selected periods of baseline and hypocapnia.

**FIGURE 4:**
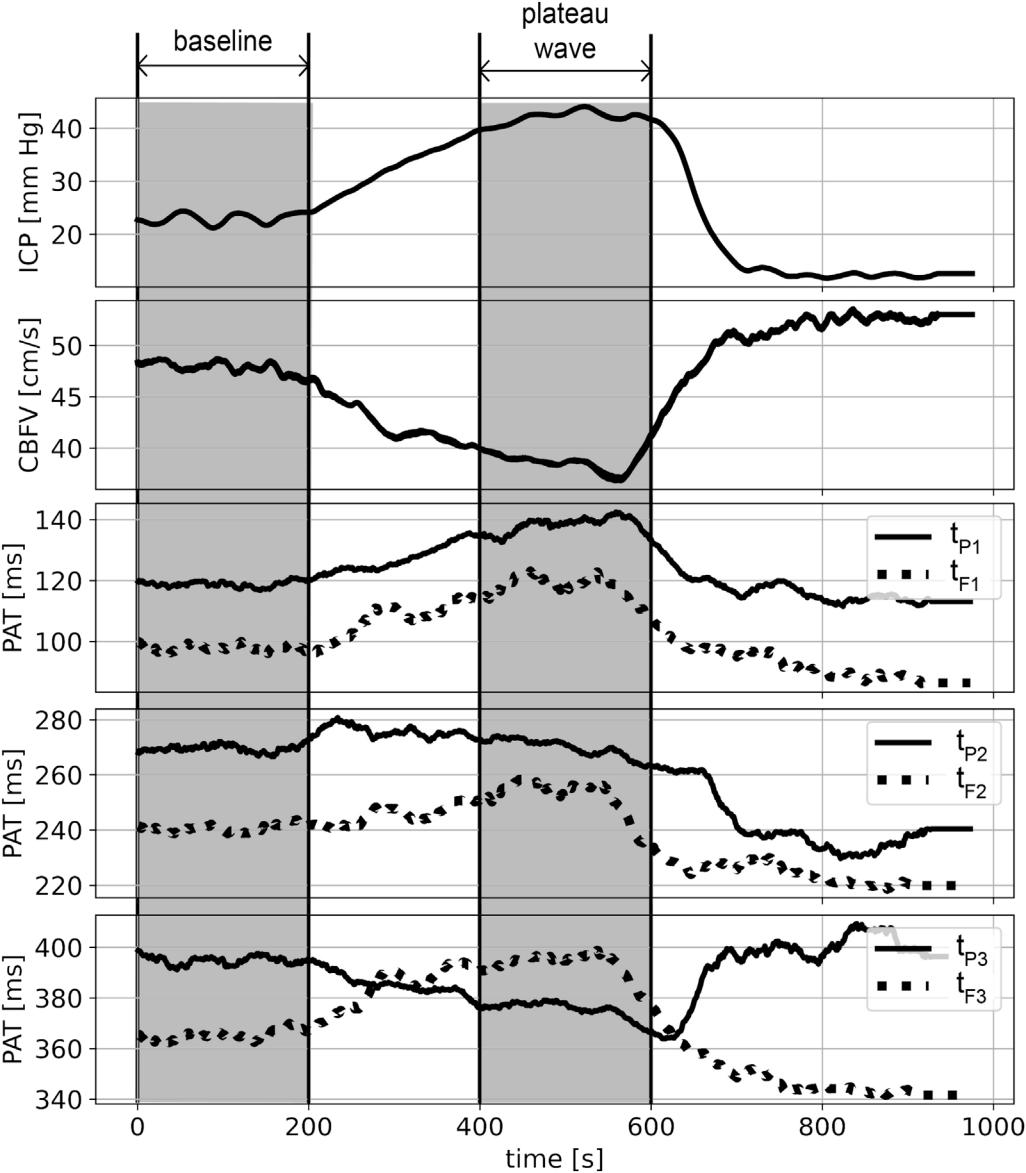
An example of smoothed (within the window of 40 pulses) recordings from a single patient during the plateau wave. From top to bottom: intracranial pressure (ICP), cerebral blood flow velocity (CBFV), peak appearance times (PATs) of the first, second, and third ICP peaks (t_P1_, t_P2_, t_P3_) and the first, second and third CBFV peaks (t_F1_, t_F2_, t_F3_). Shaded areas indicate selected periods of baseline and the flat part of the plateau wave.

ICP, CBFV (averaged over every single pulse) and PAT time courses in each recording were smoothed with a moving average filter (window length: 40 pulses, moved every single pulse). All physiological signals and PATs were plotted over time individually for each patient. Examples of such plots are presented in Figure 3 and Figure 4.

In further analysis, the mean values of ICP and CBFV peak appearance times were calculated over every baseline period and the subsequent period of hypocapnia or plateau phase in order to assess the direction of changes in each signal.

### Statistical analysis

Non-parametric tests were used for statistical analyses (assumption of normality was rejected by the Shapiro–Wilk test for the majority of variables). The distributions of calculated values of physiological signals and indices were described with the median and the upper and lower quartiles. The differences in physiological signals and indices between ICP plateau phase or hypocapnia and corresponding baselines were tested with Wilcoxon signed rank test. Spearman’s correlation coefficients were calculated individually for each patient between PATs and ICP time series. The significance level was set at 0.05 in all analyses.

## Results

### Number of peak annotations and duration of selected analysis periods

In the plateau wave group of 8 recordings, all three peaks were annotated in 11,056 ICP pulses and 9 695 CBFV pulses (8 473 common pulses). In 11 hypocapnia recordings, the peaks were annotated in 16,991 ICP pulses and 24,111 CBFV pulses (15,567 common pules). The differences in number of annotated pulses in ICP and CBFV are mainly due to signal artifacts and indistinguishability of any of three peaks in a pulse. Median length of the hypocapnia period and the baseline preceding hypocapnia were 700 s (IQR: 673–750 s) and 800 s (IQR: 800–900 s), respectively. Median length of the plateau period and the baseline before the plateau wave were 275 s (IQR: 223–470 s) and 330 s (IQR: 275–425 s), respectively.

### Changes in physiological signals

ICP decreased during hypocapnia and increased during plateau waves (see Table 1). CBFV decreased in both groups, while ABP remained stable. HR did not change between baseline and hypocapnia or between baseline and plateau waves. ICP at pre-plateau wave baseline was higher than at pre-hypocapnia baseline (*p* = 0.006).

**TABLE 1:** Medians, upper quartiles, and lower quartiles (in brackets) of physiological parameters: intracranial pressure (ICP), arterial blood pressure (ABP), cerebral blood flow velocity (CBFV) and heart rate (HR).

Parameter	Pre-hypocapnia baseline	Hypocapnia	Pre-plateau wave baseline	Plateau wave
	*n* = 11		*n* = 8	
ICP [mm Hg]	14.63 (9.10; 16.43)	9.94 (4.30; 12.10)	19.86 (17.84; 24.22)	44.14 (40.50; 46.94)
	*p* = 0.003		*p* = 0.012	
ABP [mm Hg]	76.67 (71.78; 82.19)	81.80 (74.67; 84.24)	76.56 (73.20; 84.93)	75.76 (72.50; 80.90)
	*p* = ns		*p* = ns	
CBFV [cm/s]	57.84 (51.95; 69.70)	51.11 (44.51; 55.25)	51.22 (35.53; 58.98)	39.53 (29.29; 56.73)
	*p* = 0.003		*p* = 0.025	
HR [1/min]	74.66 (69.75; 78.04)	76.69 (73.04; 80.42)	69.10 (62.66; 79.08)	69.79 (60.43; 80.97)
	*p* = ns		*p* = ns	

n–number of patients in the group; *p*–*p*-value of Wilcoxon signed rank test; ns means *p*-value greater than 0.05.

### Changes in peak appearance times

**FIGURE 5:**
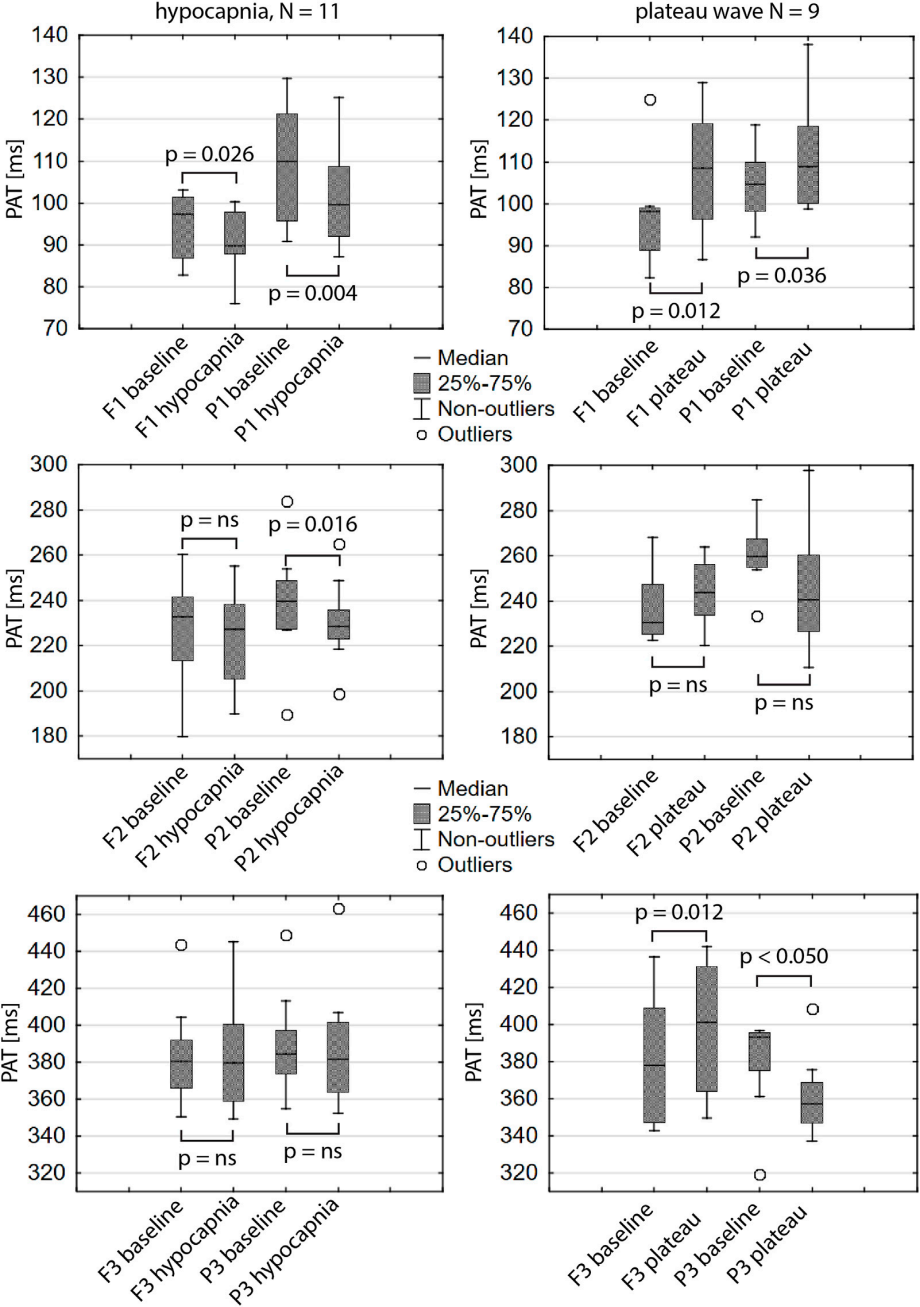
Peak appearance times (PATs) of the first, second and third peaks of intracranial pressure (P1, P2, P3) and cerebral arterial blood flow velocity (F1, F2, F3). Box-plots in the left column present PATs during hypocapnia and preceding baseline. Right column shows PATs during ICP plateau waves and corresponding baselines. N–number of patients in the group, *p*–*p*-value of Wilcoxon signed rank test, ns–*p*-values greater than 0.05.

**FIGURE 6:**
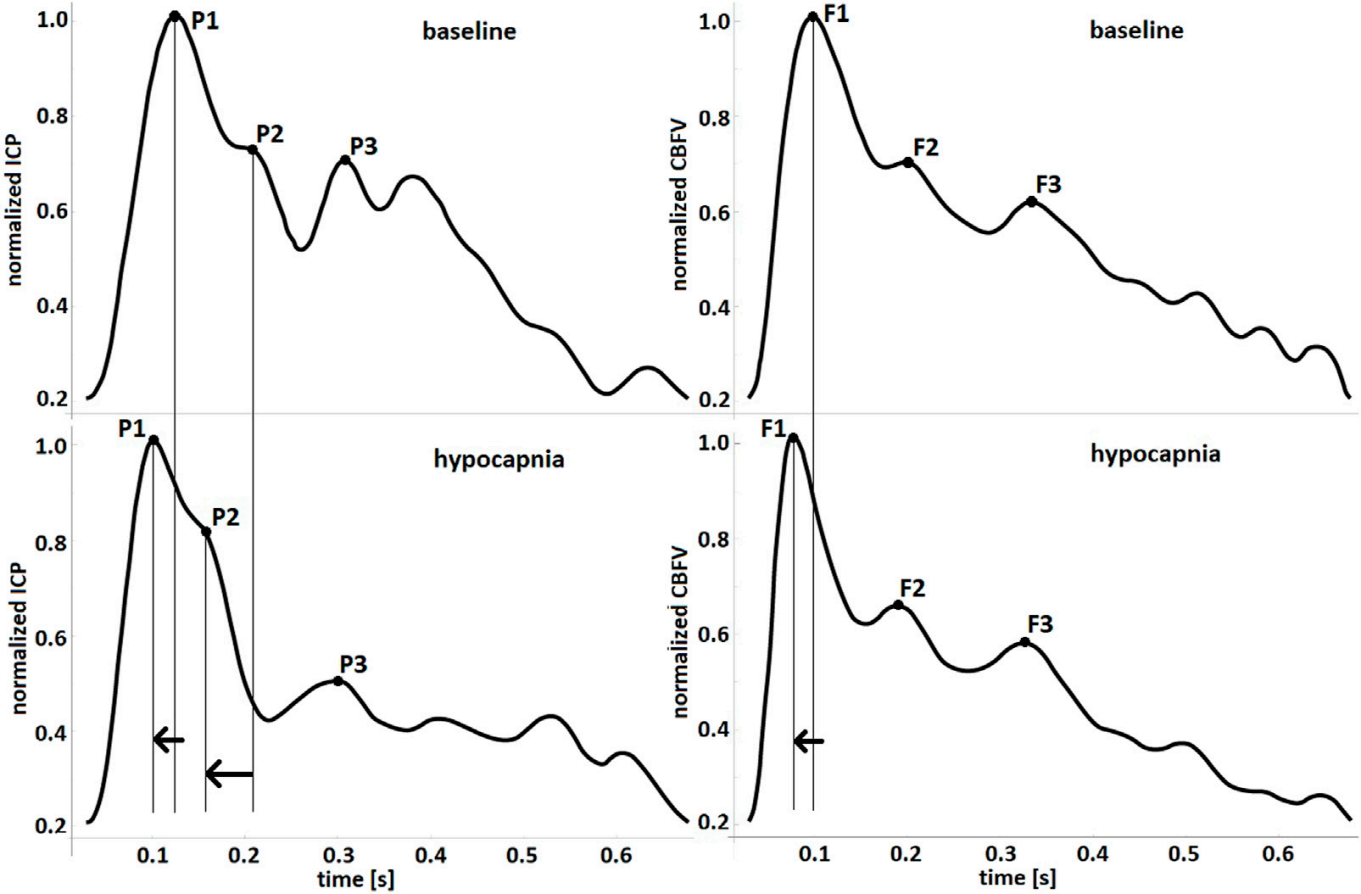
Observed changes in peak appearance times during hypocapnia presented in handpicked pulse waveforms of intracranial pressure (ICP, left-hand charts) and pulse cerebral blood flow velocity (CBFV, right-hand charts). The arrows indicate direction of changes in peak appearance times.

**FIGURE 7:**
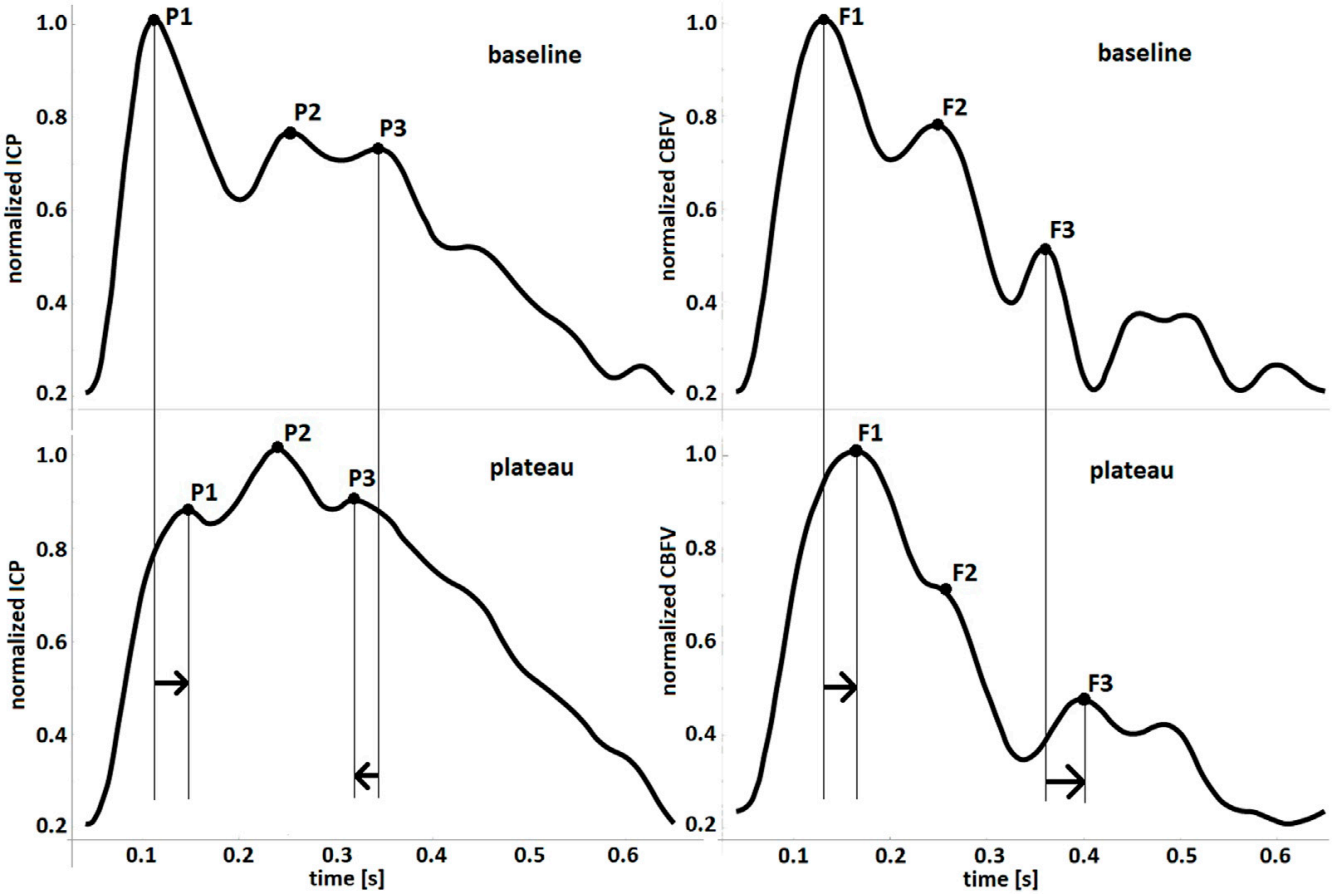
Observed changes in peak appearance times during episodes of intracranial pressure (ICP) plateau waves presented in handpicked pulse waveforms of ICP (left-hand charts) and cerebral blood flow velocity (CBFV, right-hand charts). The arrows indicate direction of changes in peak appearance times.

PAT of the first peak of ICP and CBFV got shorter during hypocapnia and longer during plateau waves (see Figure 5). During plateau waves P3 appeared earlier, whereas F3 appeared later. Second peak of ICP appeared earlier with hypocapnia. All other appearance times did not change significantly. The direction of changes (acceleration or delay) in PATs during hypocapnia and plateau waves are schematically presented in selected individual pulses in Figure 6 and Figure 7, respectively.

### Association between changes in peak appearance times and changes in mean intracranial pressure

The changes in PAT of all three peaks in the CBFV pulses were positively correlated with changes in ICP in almost all individual recordings—see Table 2. This means that during a decrease in ICP induced by hypocapnia PAT of all three peaks of CBFV pulses got shorter in all individual recordings. Opposite changes took place during an increase in ICP related to episodes of ICP plateau waves (PATs of all three peaks in CBFV pulse got longer in almost all individual recordings except for PAT of the second CBFV peak. PAT of F2 was not correlated with mean ICP in 1 out of 9 recordings (11%) during plateau waves).

**TABLE 2:** Correlation analysis of individual ICP recordings (the whole monitoring period–from the beginning of baseline to the end of hypocapnia/plateau wave). Median Spearman’s correlation coefficient (r_S_) values between mean intracranial pressure (ICP) and mean peak appearance times calculated from all statistically significant individual correlations with each group.

Group	Hypocapnia ICP⇓	Plateau wave ICP⇑
vs	mean ICP [mm Hg]	
t_P1_ [ms]	0.70 (0.25; 0.79) p_ss_ = 100%	0.77 (0.62; 0.83) p_ss_ = 75%
t_P2_ [ms]	0.65 (0.32; 0.78) p_ss_ = 100%	-0.74 (-0.86; -0.05) p_ss_ = 100%
t_P3_ [ms]	0.16 (-0.51; 0.32) p_ss_ = 100%	-0.83 (-0.94; -0.73) p_ss_ = 100%
t_F1_ [ms]	0.56 (0.38; 0.69) p_ss_ = 100%	0.79 (0.61; 0.88) p_ss_ = 100%
t_F2_ [ms]	0.32 (0.06; 0.60) p_ss_ = 100%	0.53 (0.35; 0.73) p_ss_ = 89%
t_F3_ [ms]	0.33 (-0.24; 0.56) p_ss_ = 100%	0.65 (0.55; 0.85) p_ss_ = 100%

t_pn_ appearance time of the *n*th peak of the ICP, pulse, t_Fn_–appearance time of the *n*th peak of the cerebral blood flow velocity (CBFV) pulse. Every cell contains the median of individual r_s_, values, interquartile range (in brackets) and the percentage of statistically significant correlations (p_ss_).

Similar behavior was observed for the first peak of ICP pulse—see Table 2. PAT of P1 got longer when ICP increased during plateau wave and shorter (significant correlations were found for 75% of analyzed recordings) with decreasing ICP during hypocapnia. During plateau waves there were also strong correlations between PAT of the second and third peak of ICP pulse and ICP (PATs of P2 and P3 got shorter with rising ICP). Shortening of PATs of P2 and P3 during a decrease in ICP associated with hypocapnia was less significant.

## Discussion

### Potential mechanism of changes in PATs of CBFV pulse

We observed that the first peak of CBFV pulse appears earlier during a slight drop in mean ICP induced by hypocapnia and appears later, together with F3, with a rise in mean ICP during plateau wave. As both conditions are associated with cerebral vascular changes, hypocapnia-induced vasoconstriction and plateau wave-induced vasodilation may be the factors affecting PATs of CBFV pulses. During hypocapnia, the vessels become stiffer which causes a decrease in C_a_ (compliance of the cerebral arterial bed) (Carrera et al., 2011; Ziółkowski et al., 2021a). Under normal conditions, the ABP forward wave causes an elastic expansion of the artery against the surrounding tissue. This expansion is greater if the difference between ABP and ICP is bigger. After the maximum of the systolic pressure wave passes, the artery returns to its original diameter, returning energy to the flow, which can be observed as acceleration in flow velocity. With stiffening of the vessels (such as induced by hypocapnia), the degree of artery expansion is smaller and the velocity of the forward traveling wave is greater. As a result, the artery wall transfers the energy to the flow faster (Laurent et al., 2006; Aggarwal et al., 2008; Flück et al., 2014), resulting in earlier appearance of F1.

The vascular changes that take place during ICP plateau waves are more complex. Firstly, in response to the initial sudden drop in mean ABP, cerebral vasodilation occurs to sustain cerebral blood flow at a physiological level. Along with an increase in mean ICP, it results in an increase in C_a_ (Carrera et al., 2011; Ziółkowski et al., 2021a). As the ICP plateau phase is achieved, the vasodilation is sustained until the vasoconstriction process begins (usually for several minutes), which leads to a return to the level of vascular tone preceding the plateau or to a new level of vascular tone (Rosner and Becker, 1984). As mentioned before, the arterial pressure wave propagation velocity depends on vascular stiffness. Due to vasodilation at the beginning of the ICP plateau wave, the vessels become less stiff, resulting in slower propagation of arterial pressure waveform and, as a consequence, later appearance of F1 and F3. Similar observation regarding the latency of CBFV onset was noted during cerebral vasodilation in (Kim et al., 2011).

### Potential mechanism of changes in PATs of pulse ICP

We observed that appearance time of P1 tends to change in the same direction as appearance time of F1 as they appear earlier with hypocapnia and later during plateau wave compared to baseline. As both P1 and F1 are associated with the systolic peak of the ABP pulse (Carrera et al., 2010), the mechanism of changes in PAT of P1 may also be related to the propagation velocity of the arterial pressure waveform which is modulated by the vascular stiffness—the stiffer the vessel, the higher the pressure waveform velocity and the earlier the appearance of the first peak. Opposite situation takes place during plateau wave–the vessel becomes less stiff and the pressure waveform velocity decreases resulting in later appearance of P1.

In turn, during hypocapnia PAT of P2 shortens. P2 was suggested to be associated with the maximum of the C_a_BV pulse, and the shape of the C_a_BV pulse was shown to change with mean ICP level in normal pressure hydrocephalus and in TBI patients (Carrera et al., 2010). However, the changes in C_a_BV pulse shape have not yet been well described, therefore it can only be assumed that the changes in PAT of P2 are potentially linked to the changes in C_a_BV pulse shape. Further studies are required to answer the question how these shapes of ICP and C_a_BV pulses are related to each other and why changes in PAT of P2 are significant during hypocapnia but are not clearly visible during ICP plateau waves.

The origin of P3 is still unknown; however, it was hypothesized to be associated with the shape of the C_a_BV pulse (Carrera et al., 2010) or with venous blood outflow (Czosnyka and Czosnyka, 2020). According to our unpublished observations of the relationship between the shapes of C_a_BV and ICP waveforms, the association between P3 and the shape of the C_a_BV waveform is very likely. Nevertheless, a further study on the shape of C_a_BV pulses may help to better understand the mechanism of changes in PAT of P3 and may lead to establish a new methodology for non-invasive estimation of the shape of ICP pulse.

### Relation with previous studies

Table S1 provided in the Supplementary Materials contains a synopsis-review of other studies on the morphology of intracerebral pulse waveforms. It is worth noting that most of previous research presented in the table focused on peak magnitudes rather than peak appearance times. Nevertheless, we can refer our results to previous investigations using the MOCAIP algorithm to derive morphological characteristics of ICP pulses (Hu et al., 2010b). It has been shown that under the conditions of increased ICP, the time period between P3 and P1 decreases in comparison with normal ICP levels. Therefore, our observations about the decrease in t_P3_ and increase in t_P1_ during plateau waves are in line with those previous results. It is also known that the shape of the ICP waveform changes with rising mean ICP value (Cardoso et al., 1983; Contant et al., 1995). The normal shape of ICP pulse observed while mean ICP is at a physiological level and cerebrospinal compliance is high resembles a saw-tooth with three distinct peaks whereas during significantly elevated ICP, the pulse is pathologically rounded and the peaks cannot be recognized. In our study, we observed that PAT of P3 decreases whereas PAT of P1 increases during plateau waves. This reflects the pathological deformation of ICP pulse waveform and the transition from the physiological, triphasic waveform to the pathological, rounded shape with only one distinguishable peak (P1 and P3 move toward P2, resulting in rounding of the pulsation). Such shape transformation, along with observed changes in PATs, may suggest that with increasing ICP, all ICP peaks are getting closer to each other. In recent studies conducted in monkeys, human infants and adults with the use of diffuse correlation spectroscopy (Fischer et al., 2020; Ruesch et al., 2020), the authors pointed out that the appearance time of the third peak of CBF is a relevant feature for the machine learning algorithm to estimate ICP non-invasively. We also observed that PAT of F3 appears later during the increase in ICP which suggest that analysis of the third peaks of both CBFV and CBF pulse waveforms may provide useful information on ICP changes. Furthermore, analysis of relations in individual recording between PATs and mean ICP value showed that PATs of all CBFV peaks and PAT of P1 were significantly correlated with the changes in mean ICP–these PATs changed in opposite directions during hypocapnia and ICP plateau waves. This may suggest that analysis of the appearance times might be sensitive to changes in cerebral hemodynamics and PATs of CBFV pulse may by useful as non-invasive indicators of changes in mean ICP value.

## Limitations

It is known that the distal systolic pulse amplification impacts the morphology of ABP pulse waveform (Geddes, 1991; Karamanoglu et al., 1993; Gao et al., 2016; Yao et al., 2017). The peak configuration differs between central and radial arteries and the second peak of pulse ABP may disappear in the distal compartment. Therefore, this study was performed without consideration of potential changes in PATs of ABP pulse waveform to avoid the error in comparing ABP peaks measured in the extracranial (and distant) compartment with single-compartment analysis of CBFV and ICP pulses. This study was performed in a small number of subjects and therefore the results should be treated as preliminary. Furthermore, all signals were up-sampled from 50 Hz to 200 Hz to increase their temporal resolution. The low pass filtering with cut-off frequency of 12 Hz was performed prior to analysis and may have had a minor impact on peak positions and observed changes in their appearance time. However, if this influence exists, it is systematical and fully reproducible. We did not perform the analysis of the influence of HR on PATs because in our groups HR did not change significantly between baselines and corresponding changes (hypocapnia and plateau wave). Nevertheless, in further studies on PATs we plan to investigate their relationships with HR or to perform pulse waveform normalization over time to minimize this influence. In this study we assumed that the analysis of peaks of CBVF and ICP is not affected by the state of cerebral autoregulation as the response time of cerebral autoregulation is about 5s (Zhang et al., 1998) and becomes longer in TBI patients (Czosnyka et al., 2009) while PATs are shorter than the cardiac cycle (below 1s). Moreover, we were not able to measure vasodilation or vasoconstriction directly; we assumed that vasoconstriction happens during hypocapnia and vasodilation is observed during the plateau phase of ICP plateau wave.

All the peaks of ICP and CBFV pulses were annotated manually by a single researcher, which may influence the accuracy of peaks position and may introduce an experimenter bias. In order to reduce the huge amount of work required to annotate every pulse manually, we plan to develop an artificial intelligence system to peak annotation in further studies. Only pulses with all three peaks visible in both ICP and corresponding CBFV pulses were included for analysis. In further studies, we will focus mainly (but not exclusively) on PATs of F1, F3 and P1 (as they seem to be the most significant based on our results). This will significantly reduce the number of rejected pulsations and will allow the analysis of a much larger amount of data. Furthermore, intensive care unit procedures may also impact the results. All patients were mechanically ventilated, which may influence the configuration of the peaks, especially P3, but its influence on PAT is unknown. Shape of the pulses may also be affected by the respiration phase (Foltz et al., 1990). This influence was minimized by averaging all parameters over baselines and challenges (hypocapnia/plateau waves) for group analyses and averaging over 40 pulses for individual analyses. Moreover, we had no information about the time of administration of medicines and the group was not big enough to assess the impact of medicines on the results. Finally, all subjects were patients with TBI, with TCD measured in the middle cerebral artery. Thus it is possible that presented results are specific to TBI patients and observed changes in PATs of CBFV may be specific to middle cerebral artery. Further research would be required to assess the relationship between PATs of CBFV and measurement site.

## Conclusion

Our results show that increase in ICP prolongs PATs of F1 and F3 while decrease in ICP shortens PAT of F1. Similarly, decrease in ICP shortens PATs of P1 and P2 while rising ICP during plateau wave delays the P1 and accelerates the appearance of the P3 in pulse ICP. We have also observed an association in individual recordings between PATs of all CBFV pulses, PAT of P1 and mean ICP. The results suggest that PATs of ICP and CBFV carry additional information about the state of cerebrospinal hemodynamics and PATs of CBFV pulse may potentially be used for non-invasive detection of ICP changes. Further study is needed to confirm these observations.

## Data Availability

The data analyzed in this study is subject to the following licenses/restrictions: The dataset is owned by the Addenbrooke’s Hospital, Cambridge, United Kingdom. Requests to access these datasets should be directed to mc141@medschl.cam.ac.uk.

## Abbreviations

ABP, arterial blood pressure [mm Hg]; C_a_, normalised compliance of cerebral arterial bed [cm/mm Hg]; C_a_BV, normalised cerebral arterial blood volume [cm]; CBF, cerebral blood flow [cm^3^/s]; CBFV, cerebral blood flow velocity [cm/s]; CPP, cerebral perfusion pressure [mm Hg]; Fn, *n*th peak of CBFV pulse; HR, heart rate [1/min]; t_Fn_, appearance time of the *n*th peak of CBFV pulse [ms]; ICP, intracranial pressure [mm Hg]; MOCAIP, morphological clustering and analysis of continuous intracranial pressure; PAT, peak appearance time; Pn, *n*th peak of ICP pulse; t_Pn_, appearance time of the *n*th peak of ICP pulse [ms]; TBI, traumatic brain injury.
