# Supplementary material for: Peak appearance time in pulse waveforms of intracranial pressure and cerebral blood flow velocity
Source: Front Physiol. 2023 Jan 4;13:1077966. doi: 10.3389/fphys.2022.1077966 (PMC9846027; doi:10.3389/fphys.2022.1077966)
Supplement: Supplementary file 1 [file Table1.DOCX]

Table S1. Synopsis of previous research on the subject of morphological analysis of intracranial pressure (ICP) and/or cerebral blood flow velocity (CBFV) waveforms with a short summary of observed behaviors of peak appearance times and peak heights. Pn – height of the n-th peak of the ICP pulse in reference to the diastolic value of the pulse, Fn – height of the n-th peak of the CBFV pulse in reference to the diastolic value of the pulse, |Pn| – absolute height of the n-th peak of the ICP pulse, |Fn| – absolute height of the n-th peak of the CBFV pulse, t_Pn_ – the appearance time of the n-th peak of the ICP pulse, t_Fn_ – the appearance time of the n-th peak of the CBFV pulse, V_XX_ – the height of the valley following peak XX, minXXX – the minimal value of the pulse of XXX. LVO – large vessels occlusion, TBI – traumatic brain injury, SAH – subarachnoid hemorrhage, NPH – normal pressure hydrocephalus.

| **Publication** | **Subjects** | **Experiment / state change** | **Observations on ICP peak appearance times** | **Observations on CBFV peak appearance times** | **Observations on ICP peak heights** | **Observations on CBFV peak heights** |
| --- | --- | --- | --- | --- | --- | --- |
| A. Kurji *et al.*, *Menopause*, 2006, (Kurji et al., 2006) | **Included no=25 females** premenopausal: 12 postmenopausal:  13 | **pre/post- menopausal** | – | - **t_F1_, t_F2_ and t_VF3_ do not change** in postmenopausal women,  - intersubject variances of t_F1_, t_F2_ increase after menopause | – | After menopause: - **\|F1\| decreases**  - **\|F2\|/\|F1\| increases**  - \|F2\| does not change |
| S. Aggarwal et al., *Liver Transplant.*, 2008, (Aggarwal et al., 2008) | **Included no=16** patients with acute liver failure | Standard clinical observation, **mean ICP groups: <20, 20-30, >30** | – | – | – | **\|F2\| increases with ICP, but with higher ICP decreases rapidly** |
| X. Hu *et al.*, *Physiol. Meas.*, 2010, (Hu et al., 2010a) | **Included no=63** SAH: 31 TBI: 26 others: 6 | Standard clinical observation, **CBF groups:  <=20/>20 ml/min/100 g** | - **t_P2_, t_P3_ increases** with low CBF | – | **With low CBF**: - **P3/P2 increases** - **P1 decreases** - minICP increases - P1 slope decreases  **Elevated P3** may be **associated** with **low CBF** | – |
| S. Kim *et al.*, *Neurocrit. Care*, 2011, (Kim et al., 2011) | **Included no=45**  TBI: 29 SAH: 15 NPH: 1 | Standard clinical observation | **Correlations** between MOCAIP ICP and CBFV indices, e.g.:  - **P1/P2 vs. t_F3_/( t_F3_-t_VF3_)**, correlation coefficient γ=–0.67 | | **Correlations** between MOCAIP ICP and CBFV indices, e.g.: **- P1/P2 and F1/F2**,  correlation coefficient γ=0.74 | |
| S. Asgari et al., *Neurocrit. Care*, 2011, (Asgari et al., 2011) | **Included no=4**  Females with chronic headaches | **Hypercapania** | Some not listed peak’s time-related indices change with hypercapnia | – | With hypercapnia: - **P2, P3, V_P3_** and minICP **increases** and other 50+ peak’s height-related indices change | – |
| S. Asgari *et al.*, *PLoS One*, 2012, (Asgari et al., 2012) | **Included no=19**  Chronic headaches: 5 Healthy: 14 | CO_2_ challenge test – **vascostricion/ vascodilataion** | – | Some of complex peak’s time-related indices changes with vasco-striction/dilatation | – | With vascodilatation - **F2, V_F2_, minCBFV, mean CBFV** and peaks height-related indices may **increase** |
| S. Kim, et al., *IEEE Trans. Biomed. Eng.*, 2013, (Kim et al., 2013) | **Included no=90** TBI: 44 SAH: 36 NPH: 10 | Standard clinical observation, **mean ICP groups: <15, 15-30, >30** | – | – | – | **F2/F3 ratio may increases with increasing ICP** |
| C. Dias *et al.*, *Neurocrit. Care*, 2014, (Dias et al., 2014) | **Included no=18** multiple trauma patients | **Plateau waves** | – | – | With plateau wave: - ampIitude of ICP increases - **P2/P1 increases** | – |
| C. Anile et al., *Interdiscip. Neurosurg. Adv. Tech. Case Manag.*, 2014, (Anile et al., 2014) | **Included no=20** NPH patients | **Infusion test** | – | – | During 11/20 infusion test P2 and P3 peaks increased | – |
| D. Flück *et al.*, *Front. Physiol.*, 2014, (Flück et al., 2014b) | **Included: no=29** Young: 10 Older: 19 | **Age, hypercapnia** | – | – | – | **With age:** - **\|F1\|, \|F2\|,** minCBFV **decrease** - **F2/F1 increases** With hypercapnia: - \|F1\|, \|F2\|, minCBFV may increase |
| D. Cardim *et al.*, *Neurocrit. Care*, 2017, (De Riva et al., 2012) | **Included no=36** ICP plateau waves | **ICP plateau wave** | – | – | – | **\|F1\| increases** with the plateau wave |
| M. O. Kim *et al.*, *J. Hypertens.*, 2017, (Kim et al., 2017) | **Included no=1020** apparently normal patients referred for 24-h blood pressure monitoring | – | **Reference values of t_F1_ and t_F2_ in normal subjects** were provided | – | – | – |
| S. G. Thorpe *et al.*, *PLoS One*, 2020, (Thorpe et al., 2020) | **Included no=106** LVO: 33 in-hospital controls: 33 out of hospital controls: 40 | LVO vs. normal volunteers | – | In patients with LVO the **diastolic part** of flow velocity pulse **may be extended** | – | In patients with LVO F2 and F3 peaks may be hard to recognize |
| A. Kazimierska *et al.*, *Acta Neurochir. (Wien).*, 2021, (Kazimierska et al., 2021) | **Included no=36** NPH patients | **Infusion test** | – | – | **P1/P2** **decreases** during infusion test | – |
